# DNA METHYLATION-BASED BIOLOGICAL AGING, PLASMA PROTEINS, AND INCIDENT FRAILTY AND PHYSICAL DISABILITY

**DOI:** 10.1093/geroni/igad104.2483

**Published:** 2023-12-21

**Authors:** Aung Zaw Zaw Phyo, Sara Espinoza, Anne Murray, Peter Fransquet, Jo Wrigglesworth, Robyn Woods, Joanne Ryan

**Affiliations:** Monash University, Melbourne, Australia. , Melbourne, Victoria, Australia; Cedars-Sinai Medical Center, Los Angeles, California, United States; Hennepin HealthCare and University of Minnesota, Minneapolis, Minnesota, United States; Monash University, Melbourne, Victoria, Australia; Monash University, Melbourne, Victoria, Australia; Monash University, Melbourne, Victoria, Australia; Monash University, Melbourne, Victoria, Australia

## Abstract

Decline in physical function occurs with advanced age, and biological aging may be a useful marker of decline in physical function, beyond chronological age. This study investigated the associations between DNA methylation-based biological aging (DNAm-based age acceleration (AA)) and plasma proteins and the risk of frailty (based on Fried phenotype and a frailty deficit accumulation index (FI)), and persistent physical disability (defined as loss of ability to perform one or more Katz ADL), among community-dwelling people aged ≥70 years enrolled in the ASPREE study. Five AA indices (Horvath, Hannum, Pheno, Grim, and DunedinPACE) and seven plasma proteins (including plasminogen activation inhibitor-1 (PAI-1), growth differentiation factor-15 (GDF-15), and leptin) were measured in blood from 560 participants (50.7% women). Incident health events were captured over a mean of 5.2 years. Cox proportional-hazard regression adjusted for gender, education, and cell counts. One-standard deviation (SD) increase in AA was associated with an increased risk of frailty (FI) (GrimAA: HR=1.48 (95% CI, 1.19 – 1.84), and DunedinPACE: HR=1.39 (95% CI, 1.11 – 1.73)) and physical disability (GrimAA: HR=1.78 (95% CI, 1.15 – 2.77)). One-SD increase in plasma proteins except for leptin was associated with a higher risk of frailty (both Fried phenotype and FI) (range HR: 1.26 to 2.14). In conclusion, accelerated Grim age and DunedinPACE appear as markers of physical impairments, even in initially healthy individuals. Variations in plasma protein levels might be helpful for the identification of pathophysiological pathways associated with the increased risk of physical impairment in later life.

